# Machine Learning Approaches Reveal That the Number of Tests Do Not Matter to the Prediction of Global Confirmed COVID-19 Cases

**DOI:** 10.3389/frai.2020.561801

**Published:** 2020-11-23

**Authors:** Md Hasinur Rahaman Khan, Ahmed Hossain

**Affiliations:** ^1^ Institute of Statistical Research and Training, University of Dhaka, Dhaka, Bangladesh; ^2^ Department of Public Health, North South University, Dhaka, Bangladesh

**Keywords:** COVID-19 disease, cluster analysis, machine learning, principal component analysis, regression tree

## Abstract

Coronavirus disease 2019 (COVID-19) has developed into a global pandemic, affecting every nation and territory in the world. Machine learning-based approaches are useful when trying to understand the complexity behind the spread of the disease and how to contain its spread effectively. The unsupervised learning method could be useful to evaluate the shortcomings of health facilities in areas of increased infection as well as what strategies are necessary to prevent disease spread within or outside of the country. To contribute toward the well-being of society, this paper focusses on the implementation of machine learning techniques for identifying common prevailing public health care facilities and concerns related to COVID-19 as well as attitudes to infection prevention strategies held by people from different countries concerning the current pandemic situation. Regression tree, random forest, cluster analysis and principal component machine learning techniques are used to analyze the global COVID-19 data of 133 countries obtained from the Worldometer website as of April 17, 2020. The analysis revealed that there are four major clusters among the countries. Eight countries having the highest cumulative infected cases and deaths, forming the first cluster. Seven countries, United States, Spain, Italy, France, Germany, United Kingdom, and Iran, play a vital role in explaining the 60% variation of the total variations by us of the first component characterized by all variables except for the rate variables. The remaining countries explain only 20% of the variation of the total variation by use of the second component characterized by only rate variables. Most strikingly, the analysis found that the variable number of tests by the country did not play a vital role in the prediction of the cumulative number of confirmed cases.

## 1. Introduction

The severe acute respiratory syndrome coronavirus 2 (SARS-CoV-2) is an infectious disease that first emerged in December 2019 in Wuhan, the capital of China’s Hubei province (Roosa et al., 2020). It has spread to nearly 213 countries and territories and has infected more than 2.3 million people as of April 17, 2020, has killing approximately 155,000 people worldwide (Max Roser and Ortiz-Ospina, 2020) (also see Figure 3). As of April 17, 2020, the highest crude fatality rate was observed in Belgium (nearly 485 per million), followed by Spain (nearly 435 per million), and Italy (nearly 390 per million) (Max Roser and Ortiz-Ospina, 2020). However, the highest number of deaths took place in United States (over 38,000), followed by Italy, Spain, and France. The countries most affected have conducted a large number of tests. As of April 17, 2020, the United States has conducted more than 3.7 million tests, followed by Russia (over 1.8 million), Germany (over 1.6 million), and Italy (approximately 1.3 million). The number of active cases is growing as the number of cases is growing. As of April 17, 2020, globally, nearly 67% of the total cases are active cases, and hence 23% are recovered (Max Roser and Ortiz-Ospina, 2020).

Most of the affected countries have been maintaining social distancing, closing educational institutes, offices, and markets to reduce the rate of spread; these methods have not had universal reach, however, and there are many countries where people are commuting in crowded public transport or even living in close quarters in urban slums (Hui et al., 2020). Also, in many countries, the public healthcare systems are insufficient and overburdened, and this poses a potentially dangerous threat to public health (Khan and Hossain, 2020b). According to World Bank data (World Bank, 2020), in 2015, Bangaldesh had 0.8 hospital beds per 1,000 people, India had 0.7 (2011), Pakistan had 0.6 (2012), and the United States had 2.9 (2012), whereas China had 4.2 (2012) beds per 1,000 people. It is recommended that intensive care unit (ICU) practitioners, hospital administrators, governments, and policymakers must prepare for a substantial increase in critical care bed capacity, with a focus not just on infrastructure and supplies but also on staff management (Phua et al., 2020).

**FIGURE 1:**
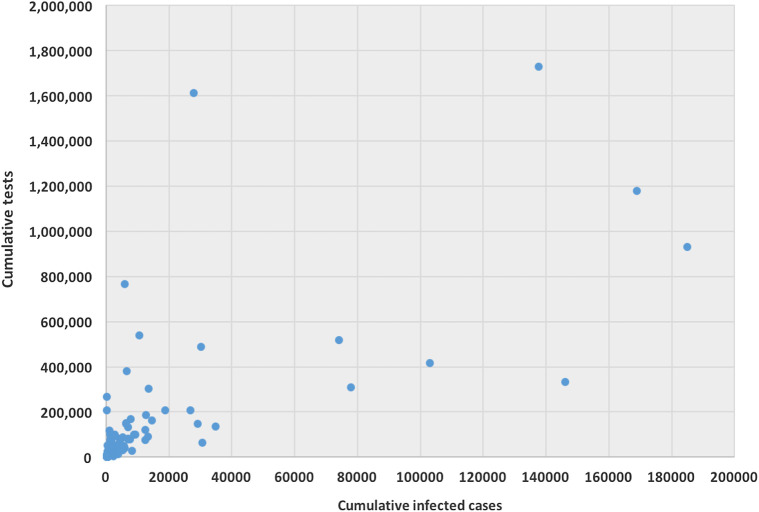
Scatter plot between cumulative tests and cumulative cases for 132 countries (except United States).

The ability for testing for COVID-19 varies from country to country. Testing ability is one of our most important tools for slowing down and reducing the spread and impact of the virus, but it is also dependent on a country’s financial capability, laboratory capacity, and access although it. Low- and middle-income countries may have to battle their COVID-19 pandemic with scarcer resources. Tests allow us to identify infected individuals, guiding the medical treatment that they receive. They also enable the isolation of those infected and the tracing and quarantining of anyone they have been in contact with (Hellewell et al., 2020). As of April 17, 2020, the United States have administered the highest no. of tests, approximately 3.4 million, which is almost 20% of global test total, followed by Germany (over 1.7 million), Russia (over 1.6 million), and Italy (approximately 1.2 million). Figure 1 is a scatter plot of the cumulative cases and cumulative tests for 132 countries. The United States was discarded for this graph since the United States have had an exceptionally high number of tests performed. We found that the correlation coefficient between these two variables for 132 countries is 0.71, which indicates a strong positive correlation; if including United States, the coefficient is 0.88, which indicates a very high positive correlation.

Artificial intelligence (AI) and machine learning expertise are needed in order to help experts within public health and epidemiology. For example, Muzammal et al. (2020) used a multi-sensor data-fusion-enabled ensemble approach for medical data. Pirbhulal et al. (2019) used machine learning tools to enable a security framework for IoT-based healthcare. AI provides a useful tool that can help in computing risk factors, classification, even drug analysis, and it can also responding to crises, according to health data specialists. Because of the increase in COVID-19 patients and the overall lack of sufficient equipment to receive all patients, difficult choices must be made. The necessary medical care is thus applied only to patients that have a higher probability of survival. Calculating the probability to survive and the effect of each feature, such as symptoms in our case, on survival probability is done using survival analysis. In the presence of massive epidemic data, the machine learning techniques help to identify the epidemic patterns so that early action can be planned to stop the spread of the virus. AI and big data can be found in a lot of applications in various fields, e.g., AI in computer science, AI in banking, AI in agriculture, and AI in healthcare. These technologies have established roles in these fields, and they currently play important roles in the global battle against the COVID-19 pandemic.

There are a number of research works where machine learning tools have been used for global and local COVID-19 data analysis. Recently, Chuanyu et al. (2020) used several machine learning tools, including elastic net, random forest, and bagged flexible discriminant analysis, for predicting the mortality risk of COVID-19 patients. This work is completely different from other COVID-19-related works since we have focused on the classification and prediction of a cumulative number of confirmed COVID-19 cases. To our knowledge, there is no work so far that has used such machine learning techniques to predict confirmed COVID-19 cases. Magdon-Ismail (2020) presented a robust data-driven machine learning analysis of the COVID-19 pandemic from its early infection dynamics. McCall (2020) discussed how artificial intelligence protects healthcare workers and helps curb the spread of COVID-19. Waiker (2020) discussed possibilities of identifying and evaluating the virus with technology, AI, and analytics. Waiker (2020) used deep learning methods to review and critically appraise published and preprint reports of prediction models for COVID-19 patients. In particular, several study works (Afshar et al., 2020; Asnaoui et al., 2020; Corman et al., 2020; Fomsgaard and Rosenstierne, 2020; Forbes, 2020; Ghoshal and Tucker, 2020; Gozes et al., 2020; Hall et al., 2020; Healthitanalytics, 2020; Hu et al., 2020a; Hu et al., 2020b; IBM, 2020; Loey et al., 2020; Maghdid et al., 2020; Narin et al., 2020; Pal et al., 2020; Pham et al., 2020; Qi et al., 2020; Rao and Vazquez, 2020; Satu et al., 2020; Sodhro et al., 2019; Yan et al., 2020; Zhang et al., 2020; Zheng et al., 2020) have used machine learning techniques, including big data techniques, to process COVID-19 data to determine the spread of disease, predict the risk of disease, and to assess the diagnosis of disease, number of incidences, and healthcare facilities.

The above studies mainly focused on the occurrence of confirmed, recovered, and fatal cases in Wuhan and the rest of the world to understand the suspected threats and plan for subsequent containment actions. To better understand and work to alleviate the COVID-19 pandemic, many papers and preprints, as outlined above, have been published online in the last 78 months. Our main purpose is to show the effectiveness of machine learning approaches to fight against the COVID-19 pandemic and review state-of-the-art solutions using these technologies. In this paper, however, we use machine learning approaches to explore whether the global cumulative number of infected people can be predicted using the data provided by Worldometer (Max Roser and Ortiz-Ospina, 2020) as of April 17, 2020. We believe that machine learning-based approaches are useful when trying to understand the complexity behind the spread of the disease and how to contain the spread of such outbreaks effectively. As the outbreak of the COVID-19 has become a worldwide pandemic, a real-time analyses of epidemiological data is needed to prepare society with better action plans to combat the disease. We also demonstrate useful approaches when using unsupervised machine learning techniques to explore the nature of propagation in different countries.

This analysis is expected to bring useful findings, as countries with poor health infrastructure, a lack of smart strategies for testing, and a lack of health care for patients could descend into a rapid spread of disease and later stages of infection. It is therefore important to use unsupervised and supervised methods to classify countries in terms of disease spread and prediction of the global number of cumulative cases of COVID-19. A number of variables are considered for this study, including the country, number of new cases, total number of active cases, total number of deaths, total number of recovered patients, total number of serious cases, total number of tests, deaths per million, cases per million, and tests per million. We are also interested in identifying what the total number of tests that are vital to predict the total number of infections for countries. We will further investigate whether the countries are clustered on the basis of these covariates. Finally, whether the total variations can be explained with some latent groups which are uncorrelated each other.

## 2. Methodology

The data used for the current study have been collected from real-time COVID-19 data from the Worldometer website (Max Roser and Ortiz-Ospina, 2020) as reported as of April 17, 2020. The Worldometer is a data repository and a free reference website that is trusted by the likes of the United Kingdom Government, Johns Hopkins CSSE, etc. For the current study, we collated the information obtained on 133 countries that have crossed the 100 number of confirmed COVID-19 cases.

**FIGURE 2:**
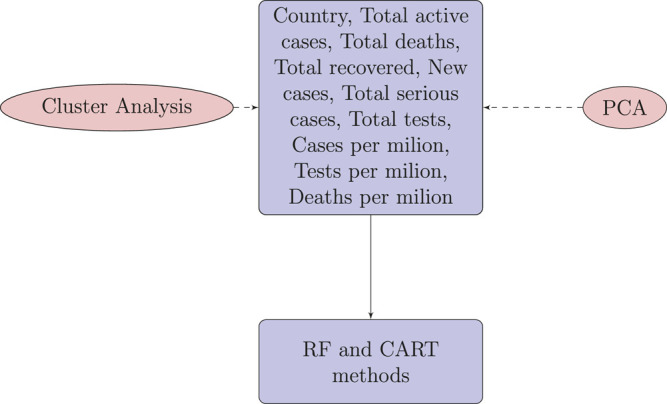
Flowchart of the methods used for COVID-19 data of 133 countries.

For each country we collected information on a total of 10 variables: the cumulative confirmed cases, new confirmed cases, cumulative deaths, cumulative recovered patients, cumulative active cases, cumulative seriously critical patients, infection rate in million, death rate in million, cumulative tests conducted, and test rate in millions. These numbers and rates are provided by the respective countries and then stored on the Worldometer website (Max Roser and Ortiz-Ospina, 2020). New confirmed cases are the confirmed cases reported on April 17, 2020. The definition of recovery and serious cases vary from country to country. According to Max Roser and Ortiz-Ospina (2020), the recovered number is not very accurate, as reports can be missing, incomplete, incorrect, and be based on different definitions or dates (or a combination of all of these) for many governments, both at the local and national level, and there may also be differences between states within the same country or counties within the same state. We considered the data that represent the rates of cases, deaths, tests per million, etc. in our analysis since these are the vital statistics that represent the proxy of the respective population size. We found a number of missing values for each variable except for the cumulative number of infected patients. There are some countries that did not provide information on the number of domestic tests performed, such as China, Kuwait, Oman, Cameroon, and Afghanistan. Before implementing any unsupervised machine learning techniques, such as principal component analysis (PCA), random forest, cluster analysis, and regression tree using the Classification And Regression Tree (CART) method Breiman et al. (1984) and the R package caret Kuhn (2020), we imputed all missing values with the Expected-Minimization algorithm technique, as suggested in Dray and Josse (2020). All 10 features were used for both PCA and cluster analysis. For CART and random forest analysis, however, the cumulative number of cases was used as a (*Y*), but all of the 10 variables were used as independent features (*X*). The pseudocode of CART and random forest methods are given below. Methods used for this study are displayed in a flowchart as displayed in Figure 2.

**Table:** 

**Algorithm 1** | CART algorithm
**Procedure CART** 1. Start at the root node 2. For each ordered value of *X*, convert it to an unordered variable $\tilde{X}$ by grouping its values in the node into a small number of intervals, **if** *X* is unordeered, **then return** $\tilde{X}=X$ 3. Perform a chi-squared test of independence for each $\tilde{X}$ variable vs. *Y* on the data in the node and compute its significance probability 4. Choose the variable $X^{\text{*}}$ associated with the $\tilde{X}$ that has the smallest significance probability 5. Find the split set $\left\{X^{\text{*}}\in S^{\text{*}}\right\}$ that minimizes the sum of gini indexes and use it to split the node into two child nodes 6. **if** a stopping criteria is reached, **then return** exit Otherwise, apply steps 2–5 to each child node 7. Prune the tree with the CART method

**Algorithm 2** | Random forest algorithm
**Procedure RF** 1. Randomly select *M* features from the feature set. 2. For each *X* in *M*, *a*. Calculate the information gain $\begin{array}{c} Gain\left(t,X\right)=E\left(t\right)-E\left(t,X\right)\\ E\left(t\right)=\overset{c}{\underset{i=1}{\sum }}-P_{i}{\text{log}}_{2}P_{i}\\ E\left(t,X\right)=\underset{c\in X}{\sum }P\left(c\right)E\left(c\right), \end{array}$ Where E(t) is the entropy of the two classes, and E(t,X) is the entropy of feature *X* *b*. Select the node *d* that has the highest information gain *c*. Split the node into sub-nodes *d*. Repeat steps *a*, *b*, and *c* to construct the tree until the minimum number of samples required to split is reached 3. Repeat steps 1 and 2 for *N* times to build forest of *N* trees

## 3. Analysis

**FIGURE 3:**
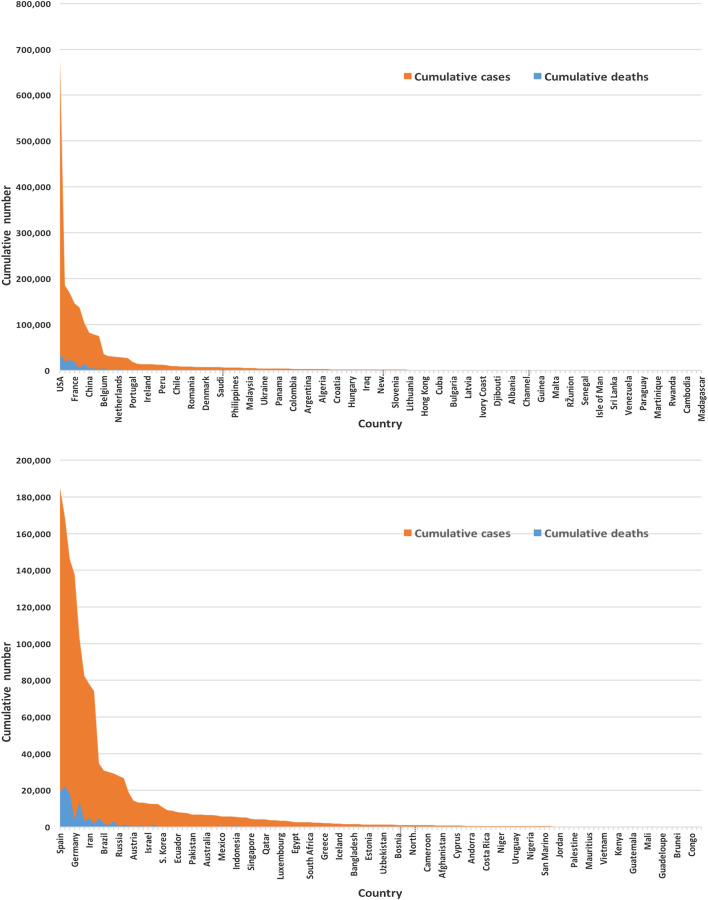
Global infected cases and deaths of COVID-19 for 133 countries **(upper panel)** and without the United States **(lower panel)** as of April 17, 2020.

Figure 3 displays of most of the COVID-19 cases and deaths are from the United States and European countries. We found that the United States and European countries, such as Germany, Russia, Italy, Spain, the United Kingdom, and France, administered a very high number of tests. The average number of tests among 133 countries is found to be nearly 156,500. The United States performed the highest at 3,398,140 and San Mario the lowest at 846 tests as of April 17, 2020.

**FIGURE 4:**
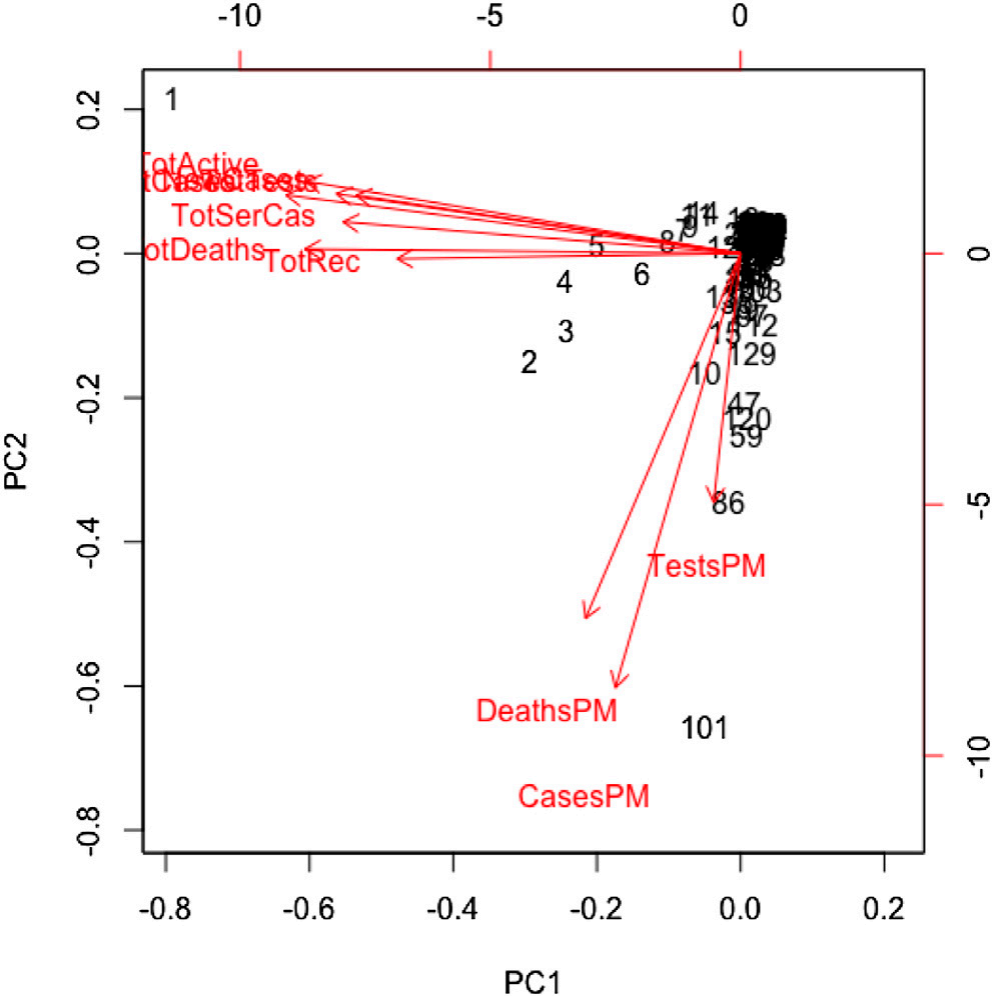
Principal component analysis results for global COVID-19 data of 133 countries.

All variables except for the country are correlated in this study. We standardized the data and imputed the missing value use of the Expectation-Maximization (EM) algorithm, according to (Dray and Josse, 2020), prior to performing the principal component analysis. We found the principal components through orthogonal transformation by converting the 10 correlated variables of the 133 countries into a set of values that are linearly uncorrelated variables. This exploratory data analysis is useful for making predictive models. This unsupervized machine learning technique will give the patterns of similarity in the countries and those orthogonal variables found. Figure 4 shows such pattern where the first two principal components are displayed. We found that most of the variance (80%) is explained by the first two principal components.

The main results are reflected in the graph of the scores in Figure 4, where we show the countries in the axes formed by the first two principal components. The cloud of individual points is centered at the origin to facilitate the data analysis. The first principal component is characterized by the variables: cumulative infected cases, cumulative deaths, active cases, cumulative recovered cases, cumulative serious cases, new cases, and cumulative tests. The countries that are vital to explaining the 60% variation of total variations by the first component include the United States, Spain, Italy, France, Germany, the United Kingdom, and Iran. The second principal component is characterized by the remaining variables: rate of deaths, rate of infected cases, and rate of tests per million. A country's population size is the proxy of these rates, playing a vital role in the second principal component, which explains 20% of the total variations.

**FIGURE 5:**
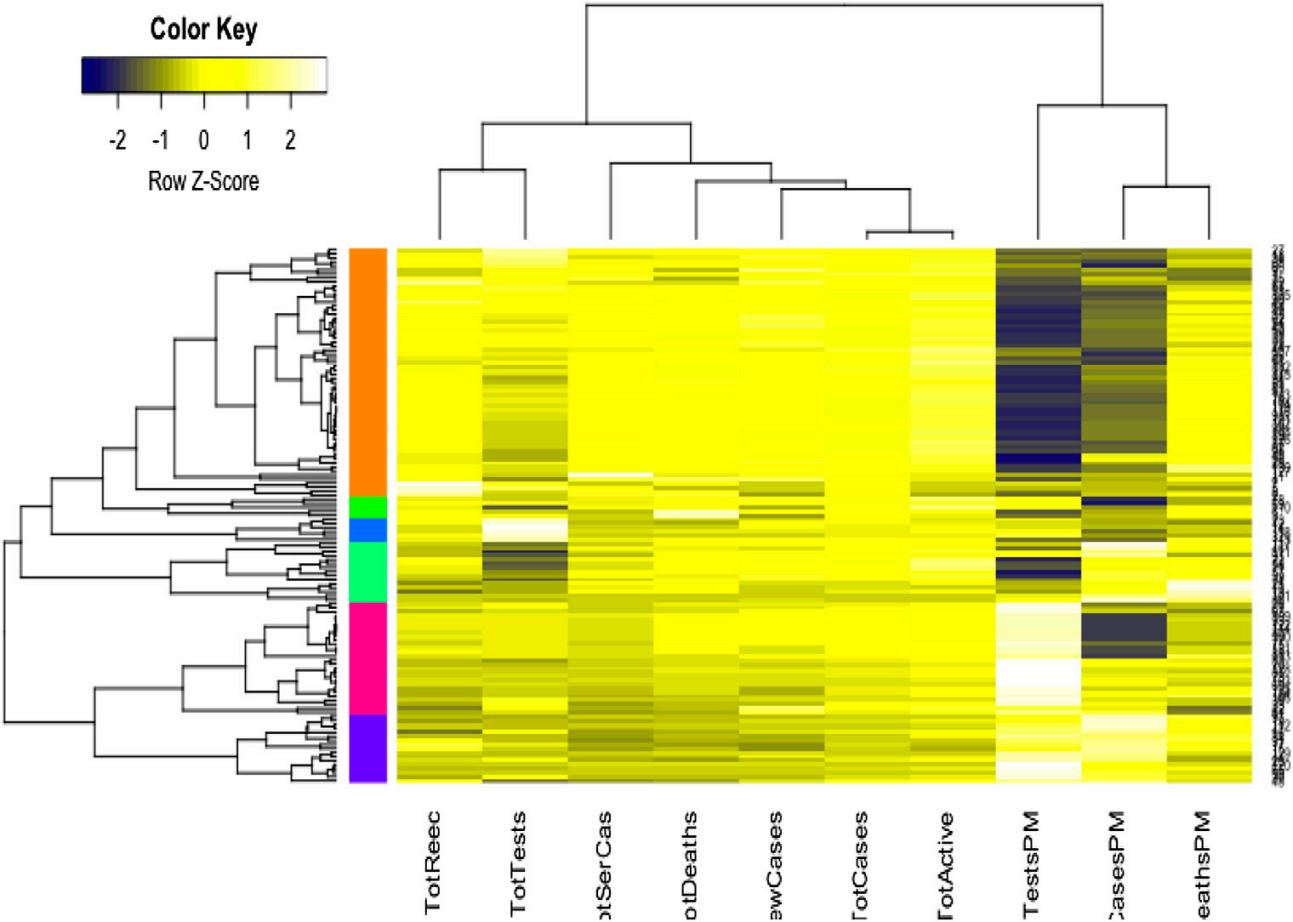
Cluster analysis results for global COVID-19 data of 133 countries.

We used the cluster analysis technique for the imputed and standardized data, as used in the principal component analysis. The heatmap of the hierarchical cluster analysis, as shown in Figure 5, reveals that there are two clusters among the variables and four clusters among the countries. Three rate variables together–tests, cases, and deaths per million form one cluster while the remaining seven variables together form the second cluster. It is mentioned that the rate variables under the first cluster together address the population number. Population is a significant factor when assessing a country’s COVID-19 response. However, we observed four major clusters among the countries.

Table 1 shows the full list of the clusters. The first cluster contains all the countries that contributed to the first principal component’s variation in the PCA analysis along with China. The PCA also suggests that we validate this clustering because the heatmap in Figure 5 reveals that these countries are clustered based on the maximum variation directed by the all seven variables. It is observed, from the data collected, that these nine countries were the most affected countries. Additionally, the economic conditions and medical facilities of these countries are among the best in the world. The second cluster contains 43 countries which are clustered according to all variables except for the test and case rates per million. Most of the 43 countries are middle income countries and have moderated health facilities. The third cluster consists of 14 countries that are clustered based on all variables other than death rate per million. These countries have much fewer deaths. These 14 countries are rich and may have well-developed health facilities available. The final cluster consists of the highest number, 68 countries, and these are clustered mainly based on the test and case rates variable, though other variables were also used in this study. Most of these 68 countries are poor, and they may thus have very poor conditions for treatment and healthcare facilities.

**TABLE 1:** Cluster-wize country lists for 133 countries.

Cluster	Country
Cluster 1 (*n* = 8)	United States, Spain, Italy, France, Germany, United Kingdom, China, Iran
Cluster 2 (*n* = 43)	Afghanistan, Australia, Austria, Belarus, Brazil, Brunei, Burkina Faso, Cameroon, Canada, Congo, Cyprus, Czechia, Denmark, Diamond Princess, DRC, Estonia, Finland, Guadeloupe, Guinea, Hong Kong, Israel, Ivory coast, Kuwait, Latvia, Lithuania, Madagascar, Mali, Martinique, Netherlands, New Zealand, Norway, Oman, Portugal, Qatar, Russia, Runion, S. Korea, Senegal, Singapore, Slovenia, Sweden, Turkey, Venezuela
Cluster 3 (*n* = 14)	Andorra, Bahrain, Belgium, Channel Islands, Faeroe Islands, Gibraltar, Iceland, Ireland, Isle of Man, Luxembourg, Malta, San Marino, Switzerland, UAE
Cluster 4 (*n* = 68)	Albania, Algeria, Argentina, Armenia, Azerbaijan, Bangladesh, Bolivia, Bosnia and Herzegovina, Bulgaria, Cambodia, Chile, Colombia, Costa Rica, Croatia, Cuba, Djibouti, Dominican Republic, Ecuador, Egypt, El Salvador, Georgia, Ghana, Greece, Guatemala, Honduras, Hungary, India, Indonesia, Iraq, Jamaica, Japan, Jordan, Kazakhstan, Kenya, Kyrgyzstan, Lebanon, Malaysia, Mauritius, Mayotte, Mexico, Moldova, Montenegro, Morocco, Niger, Nigeria, North Macedonia, Pakistan, Palestine, Panama, Paraguay, Peru, Philippines, Poland, Romania, Rwanda, Saudi Arabia, Serbia, Slovakia, South Africa, Sri Lanka, Taiwan, Thailand, Trinidad and Tobago, Tunisia, Ukraine, Uruguay, Uzbekistan, Vietnam

We implemented the regression tree using CART to predict the cumulative number of infected people. The main purpose of implementing the regression tree is to see whether the global cumulative number of infected people can be predicted accurately using the 10 variables in this study. Results are presented in Table 2, which shows the weights, including their percentage of importance, for all 10 variables. It revealed from the results that country and cumulative active cases appeared to be the most important variables to predict the cumulative number of infected people, and these were followed by the cumulative deaths, cumulative recovered cases, new case, and cumulative serious cases. Most strikingly, however, we found that the cumulative tests appeared as one of the most unimportant variables to predict the cumulative number of infections.

**TABLE 2:** Importance of variables by regression tree and random forest.

	Regression tree	Random forest
Variable names	Percentage of importance (weights)	
Country	25 (454.2)	19.1 (31.4)
Total active cases	24 (421.8)	16.1 (26.5)
Total deaths	16 (332.7)	15.7 (25.8)
Total recovered	14 (255.2)	13.2 (21.7)
New cases	10 (174.7)	14.0 (23.0)
Total serious cases	8 (149.0)	13.3 (21.9)
Total tests	1 (17.4)	7.2 (11.9)
Cases per million	1 (17.3)	0.3 (0.5)
Tests per million	0 (1.0)	0.2 (0.4)
Deaths per million	0 (0.0)	0.9 (1.4)
RMSLE	0.339	0.287

We also implemented the random forest to predict the cumulative number of infected people. The random forest is a model made up of many decision trees that are then transformed into a single ensemble model. This model uses two key concepts–random sampling of training data points when building trees and random subsets of features considered when splitting nodes. The decision tree is prone to overfitting when we do not limit the maximum depth, and this is due to its unlimited flexibility. As an alternative to limiting the depth of the tree, which reduces variance and increases bias is the random forest. Results are presented in Table 2, which shows the weights, including their percentage of importance, of all 10 variables. The weight is the total decrease in node impurities, measured by the Gini Index from splitting the variable, averaged over all trees. We found very similar results for the regression tree using CART. That is, country and cumulative active cases appeared to be the most important variables with which to predict the cumulative number of infected people. And the cumulative tests appeared to be one of the unimportant variables with which to predict the cumulative number of infections. This is a striking finding obtained by using both the CART and random forest methods. We have not found any other studies that have obtained a similar result.

The prediction accuracy for both methods, regression tree and random forest, has been measured with the root mean square log error (RMSLE). The RMSLE is calculated as

$$\text{RMSLE }\left({\hat{y}}_{i},y_{i}\right)=\sqrt{\frac{1}{n}\underset{i=1}{\overset{n}{\sum }}{\left[\text{log}\left({\hat{y}}_{i}+1\right)-\text{log}\left(y_{i}+1\right)\right]}^{2}}$$

where *n* is the total number of observations, ${\hat{y}}_{i}$ is the predicted value, and $y_{i}$ is the actual value for the *i*th cases. Here, $\text{log}\left(y_{i}\right)$ is the natural logarithm of $y_{i}$. Table 2 shows that the random forest method can predict more efficiently than the regression tree method. This suggests that random forest, as expected, is better when predicting the global cumulative cases of COVID-19.

## 4. Discussions and Conclusions

In this paper, we demonstrated how to implement the basic machine learning techniques–principal component, cluster analysis, and regression tree to analyze global COVID-19 data that was extracted from the Worldometer website (Max Roser and Ortiz-Ospina, 2020) as of April 17, 2020. We considered 10 variables for each of the 133 countries. Through use of PCA analysis found that there are two latent variables that are characterized by the 10 variables we considered. The first principal component explains the 60% variation of the total variations, and this is characterized mainly by seven variables. These are the total infected cases, deaths, active cases, recovered cases, serious cases, new cases, and total tests. The majority of the total variations is made up of all variables except for the rate variables. The remaining three variables—case, death, and test rates (measured in per million)—characterize the second principal component, which accounts for the 20% variation of the total variations. The latent factor behind this appears to be the country’s population size, as all these three variables representing their population size. None of the populations (nor the population densities) of the 133 countries are. We believe that country’s population size or indirectly the associated population density is responsible for the 20% variation of the total variations.

The cluster analysis found four major clusters among the countries but two clusters among the 11 variables. The analysis reveals that the countries are clustered based on the variation among the variables. We found that the eight countries that have the highest number of cases form a cluster, while 43 countries form another cluster based on all the variables except for the case and test rates. The eight countries are the United States, Spain, Italy, France, Germany, the United Kingdom, China, and Iran, and they are all homogeneous in term of cumulative cases, deaths, active cases, and tests. Most of them were/are the epicenter of the pandemic. However, we found that 14 countries with very low rates of death form one cluster and 68 countries with higher test and case rates, along with the significant effect of the other eight variables, form the fourth cluster. Countries and territories with low death rates include Bahrain, Belgium, Channel Islands, Faeroe Islands, Gibraltar, Iceland, Ireland, Isle of Man, Luxembourg, Malta, San Marino, Switzerland, and the UAE.

We found from both the regression tree and random forest analyses that country, total active cases, total deaths, total recovered cases, new cases, and total serious cases are very important variables with which to predict the cumulative number of cases. The number of tests (including the three rate variables) is not an important variable. As stated, global data analysis indicates that the cumulative number of tests is not significant when predicting cumulative cases, but it is quite important to consider a specific country in terms of situation and context. Besides, the policies on testing differ from country to country, region to region, or even city to city. It mainly depends on what stage a specific country or community has reached in terms of the pandemic curve or the level of preparedness in terms of lab facilities, lab staff, sample collection strategies, etc. When resources are limited and the healthcare system is overloaded, widespread testing, such as that suggested by the World Health Organization (WHO), may not be implemented. This is a reality for many of the low- and middle-income countries on our list of 133. The number of tests is important for many countries to limit the spread in the early stages (or even in any stage of spread), as this affects the ability to identify cases and isolate them and their contacts. However, global COVID-19 data analysis results reveal that cumulative tests are not at all important determinants with which to predict the cumulative number of tests for the country.

The world grapples with the containment of the COVID-19 outbreak, and countries are trying to reduce virus spread by performing tests for detecting and then isolating the infected people and quarantining the susceptible people. Besides, continuing the lockdown and social distancing is expected to help in reducing the spread considerably. However, this paper found that the countries are clustered with respect to underlying effects of the covariates, though the countries are fighting independently against this virus war. Similarly, variables related to rates form a cluster together while other variables form another cluster. Most strikingly, we found that the cumulative tests appeared as an unimportant variable when predicting the cumulative number of infected people.

This study was conducted to assess how the countries are clustered in terms of the covariates. Implementation of unsupervized and supervised methods revealed that the classification of countries is important, as it might help when analyzing the spread of disease and predicting the global cumulative cases of COVID-19. However, the countries in each cluster might have different strategies and policies with which to control the epidemic outbreak. They should all depend on data-dependent strategies, such as tracing and tracking the reproduction number of COVID-19, when developing methods with which to control the outbreak. Some early studies with data from Wuhan revealed the importance of exploring the reproduction number of COVID-19 (Kenji et al., 2020; Qun et al., 2020). Also, countries within the cluster need to evaluate several health facilities and their preparedness. So, the unsupervised learning could be useful when learning about the shortcomings of health facilities in the groups where infection is higher and when assessing what strategies are necessary when trying to prevent the spread of infection within or outside the country. Besides, classification and grouping based on the underlying latent feature could be useful to countries when trying to control the epidemic outbreaks through common remedial measures.

We used the CART and random forest methods, although random forest has better predictive power and accuracy than a single CART model due to the lower variance exhibited by the random forest. Our main goal was to know whether the independent features are significantly associated with the dependent variable “cumulative cases” rather than predictive accuracy. However, the CART has advantages: the rules are easily interpretable and it offers automatic handling of variable selection, missing values, outliers, local effect modeling, variable interaction, and non-linear relationships. Although the definition of recovery may vary from country to country, this study has used the number of recovered people, without knowing the actual definition of the recovery from COVID-19, for the respective country. This is a limitation of this study. One of the future directions could be the comparison of results of regression tree using CART and random forest methods with other machine learning counterparts, such as the support vector machine (SVM) and deep learning methods.

## Data Availability

Publicly available datasets were analysed for this study. The data used for the current study has been collected from the real time COVID-19 data from the Worldometer website (Max Roser and Ortiz-Ospina, 2020) until April 17, 2020. The working data set used for this study has been submitted to the journal as additional supporting file.
